# Stress and Depressive and Anxiety Symptoms in the General Population and in SARS-CoV-2-Infected Patients—Findings from a Population-Based Three-Wave Study

**DOI:** 10.3390/jcm12196240

**Published:** 2023-09-27

**Authors:** Hannah Wallis, Melanie Elgner, Marisa Schurr, Katrin Elisabeth Giel, Peter Martus, Gregor Paul, Jan Steffen Jürgensen, Christine Allwang, Rafael Mikolajczyk, Annette Galante-Gottschalk, Stefan Ehehalt, Florian Junne, Marius Binneböse

**Affiliations:** 1 Department of Psychosomatic Medicine and Psychotherapy, University Hospital Magdeburg, 39120 Magdeburg, Germany; melanie.elgner@med.ovgu.de (M.E.); marius.binneboese@med.ovgu.de (M.B.); 2 German Center for Mental Health (DZPG), Site Jena-Magdeburg-Halle, 07745 Jena, Germany; 3 Department of Psychosomatic Medicine and Psychotherapy, Medical University Hospital Tuebingen, 72076 Tuebingen, Germany; 4 Institute for Medical Biometrics and Clinical Epidemiology, University Hospital Tuebingen, 72076 Tuebingen, Germany; peter.martus@med.uni-tuebingen.de; 5 Klinikum Stuttgart, 70174 Stuttgart, Germany; 6 Department of Psychosomatic Medicine and Psychotherapy, Klinikum Rechts der Isar, Technical University of Munich, 80333 Munich, Germany; christine.allwang@mri.tum.de; 7 Institute for Medical Epidemiology, Biometrics and Informatics, Interdisciplinary Center for Health Sciences, Medical School, Martin-Luther University Halle-Wittenberg, 06120 Halle (Saale), Germany; 8 Public Health Department of Stuttgart, 70176 Stuttgart, Germany; annette.galante-gottschalk@stuttgart.de (A.G.-G.); stefan.ehehalt@stuttgart.de (S.E.)

**Keywords:** COVID-19, pandemic, mental health, stress, depressive symptoms, anxiety

## Abstract

*Objective*: Understanding factors that impaired mental health during the COVID-19 pandemic is extremely relevant in order to mitigate long-term consequences of the pandemic and to promote resilience in future crises. *Method*: Data were collected in southern Germany in a population-based survey study (CoKoS) with three times of measurement in May 2020, November 2020 and July 2021. Predictors of depressive and anxiety symptoms were measured with a short version of the Patient Health Questionnaire (PHQ-4) in the general population (*N* = 758) and individuals who were infected with SARS-CoV-2 in the beginning of the pandemic (*N* = 412). We investigated differences between both samples and how stress components (*worry*, *tension*, *demands and joy*) measured with the Perceived Stress Questionnaire (PSQ) varied with depressive and anxiety symptoms over time. Three linear mixed models (GLMMs) were fitted to predict the PHQ-4 stepwise, including sociodemographic variables and stress (PSQ). *Results*: Depressive and anxiety symptoms increased from May 2020 to November 2020 and remained stable until July 2021. There were no differences between people with SARS-CoV-2 infection and the general population. Those with a pre-existing disease and lower education reported higher levels of depressive and anxiety symptoms. Stress explained a substantial fraction of variance in depressive and anxiety symptoms. The stress component *worry* emerged as the strongest predictor of depressive and anxiety symptoms, whereas *joy* seemed to buffer these symptoms. *Conclusions*: The results suggest that mitigating people’s worry and increasing joy may promote resilience in future crises. Future studies should assess mental health interventions targeted at vulnerable groups, such as those with lower socioeconomic status and poorer health.

## 1. Introduction

The COVID-19 pandemic is a health crisis that has severely affected people and whole health care systems. Particularly in the first waves of the pandemic in 2020 and 2021, health care professionals were confronted with questions on how the pandemic affected people’s mental health. Besides changes in people’s daily lives, such as, for example, contact regulations or cancellation of social events, vulnerability and fear for one’s health or the health of others were debated as potential stressors with negative effects on mental health [1]. Moreover, discussion started on whether the SARS-CoV-2 infection itself is associated with symptoms such as depression and anxiety [2,3].

Meanwhile, early studies showed that the prevalence of elevated depressive symptoms increased during the pandemic in the general population [4,5,6,7], such as a study of US adults reporting increases from 27.8% in 2020 to 32.8% in 2021 in the general population [8]. However, only a few studies focused on depressive symptoms in people with SARS-CoV-2 infections during the further course of the pandemic and people without an infection followed prospectively over the same time. Therefore, the question whether the increase in depressive symptoms is a specific symptom accompanying a SARS-CoV-2 infection or is generally related to the pandemic and the corresponding stressors remains a relevant research topic. 

This study aims to contribute to this research by investigating symptoms of depression and anxiety measured with the short version of the Patients Health Questionnaire (PHQ-4) in both a sample of the general population representative for a city in southern Germany and in a sample of people with proven SARS-CoV-2 infection in the same region. In both samples, we focused on how different stress components (*worry*, *tension*, *demands and joy*) measured with the Perceived Stress Questionnaire (PSQ) varied with depression and anxiety symptoms over time.

### 1.1. Depressive and Anxiety Symptoms in the COVID-19 Pandemic 

Mental health clearly declined during the COVID-19 pandemic in the general population, as supported by various studies and recognized by *The Lancet* Commission on lessons for the future from the COVID-19 pandemic [9]. For subjective well-being and general mental health, these changes during the first year of the pandemic were small [10].

However, a systematic review and meta-analysis reported elevated ratings of depression and mood disorder symptoms from May to July 2020 compared to pre-pandemic measures [4,5]. Especially higher daily infection rates and reductions in human mobility were associated with an increased prevalence of major depressive disorder and anxiety disorders worldwide in 2020 (results from 73 studies [5]). Moreover, particularly those with lower socioeconomic status and poorer mental health reported lower well-being and mental health than those with more socioeconomic resources [9]. A study from Germany indicated that increases in fear, anxiety and depressive symptoms were higher in individuals with a pre-existing mental disorder [6]. 

Based on these studies, it seems interesting which inner-psychic processes, such as experiencing stress, predict the increase in depressive symptoms, particularly in vulnerable groups with pre-existing mental or physical diseases. This focus on inner psychological processes on the one hand and vulnerable groups on the other hand allows practitioners to adapt their prevention and therapy to the needs of people suffering the most in the pandemic. 

### 1.2. The Role of Stress as a Predictor of Depressive Symptoms in the COVID-19 Pandemic

The pandemic of COVID-19 as a global health, economic and social crisis built up stress in individuals. The Transactional Theory of Stress (TTS) is a well-studied theory [11,12] investigating how individuals handle stressful situations. Following Lazarus and colleagues, reacting to a stressful situation (e.g., the COVID-19 pandemic) with emotions such as worry and fear bears risks for individuals’ health, whereas a more positive outlook might buffer mental health symptoms. 

Similar to the increases in depressive symptoms during the pandemic, various studies indicate increases in people’s perceived stress during the pandemic [2,13,14]. A German study using the Perceived Stress Questionnaire (PSQ) reported increases in the stress components *worry* and *tension* and a lack of *joy* but a decrease in *demands* during the first lockdown in Germany, compared to stress before the pandemic [15]. Studies suggest that the perception of threat to mental and physical health increased during the pandemic [16], which might particularly result in higher stress in the form of worry.

Based on this theoretical background and empirical evidence, we were interested in perceived stress and its components *worry*, *tension*, *demands and joy* as predictors of depressive symptoms during the pandemic. 

### 1.3. Depressive Symptoms among SARS-CoV-2-Infected versus General Population 

A retrospective pre–post cohort study from the US included 3690 adult patients diagnosed with SARS-CoV-2 and matched those with controls without an infection, measuring global mental- and physical-health-related quality of life [17]. Compared to before the infection, SARS-CoV-2 patients reported a reduction in their mental health, and compared to the control group, mental health was worse following a SARS-CoV-2 infection [17]. The results from a cohort study from Norway including 70,000 adults with *N* = 774 participants that self-reported to be infected with SARS-CoV-2 indicated only a low excess risk of anxiety and depression after a respective infection [18]. However, the risk of depression seemed to be higher for participants with a severe infection, compared to mild infections.

To sum up, so far, the results regarding the association between depressive symptoms and SARS-CoV-2 infection seem ambiguous. There are indications for associations between a severe SARS-CoV-2 infection and higher depressive symptoms. However, whereas some cohort studies report higher mental health symptoms after a SARS-CoV-2 infection, others indicate lower excess risks of depressive symptoms. Moreover, it seems unclear how the infection itself is associated with depressive symptoms because only a few studies provide samples with proven infection and comparable population-based control groups. In addition, many studies report a sharp increase in stress during pandemic times. However, respective predictions of depressive symptoms seem to have been little investigated to date. Our longitudinal study aims to further investigate how trajectories and predictors of depressive symptoms and stress develop in people with a proven SARS-CoV-2 infection versus non-infected individuals.

## 2. Methods

### 2.1. Data Analysis

We used SPSS, R (version v3.6.0, http://www.r-project.org (accessed on 1 August 2022)) and lme4 [19] to perform a generalized mixed effects analysis to investigate the trajectories of anxiety and depression of the general population (*N* = 758) and in persons with SARS-CoV-2 infections (*N* = 412) from May 2020 (T0) to November 2020 (T1) and July 2021 (T2). We fitted three linear mixed models (estimated using ML and nloptwrap optimizer) to predict the *PHQ-4*. In *model 1*, we predict the PHQ-4 with *time*, *sample*, *education* and *pre-existing disease* (formula: PHQ-4 ~ (time + I (time^2^)) + sample × time + education + disease). In addition to these variables, we also add *general stress* as predictor in the form of the PSQ in *model 2*. In *model 3*, we integrate the different subcategories of stress (PSQ) in the form of *worry*, *tension*, *demands and joy* as predictors (instead of general stress). All three models include time and the ID (Pseudonym) as random effects (formula: ~1 + time|Pseudonym). A non-significant Little’s test (MCAR test [20]) indicated that data are missing completely at random (χ^2^ (675) = 645.25, *p* = 0.789).

### 2.2. Sample and Procedure

We used data collected as part of a population-based longitudinal study with three times of measurement, examining differences between the mental health of people with a SARS-CoV-2 infection in Stuttgart, a large city in southern Germany, and a sample that was representative of the general population in that city [7].

Data on the non-infected general population were derived from a probability sample, which was drawn by the residents’ registration office and was representative of the adult population living in Stuttgart. In total, 4400 adult members were invited to participate in the study via postal letters.

Data for the participants with a SARS-CoV-2 infection were collected in cooperation with the local public health authority, contacting adults who officially registered their infection until April 2020. All 1267 adult registered individuals with proven SARS-CoV-2 infection were invited to participate in this study by the public health authority. Baseline measurement took place shortly after the first registered SARS-CoV-2 infection in Germany (27 January 2020).

**Figure 1:**
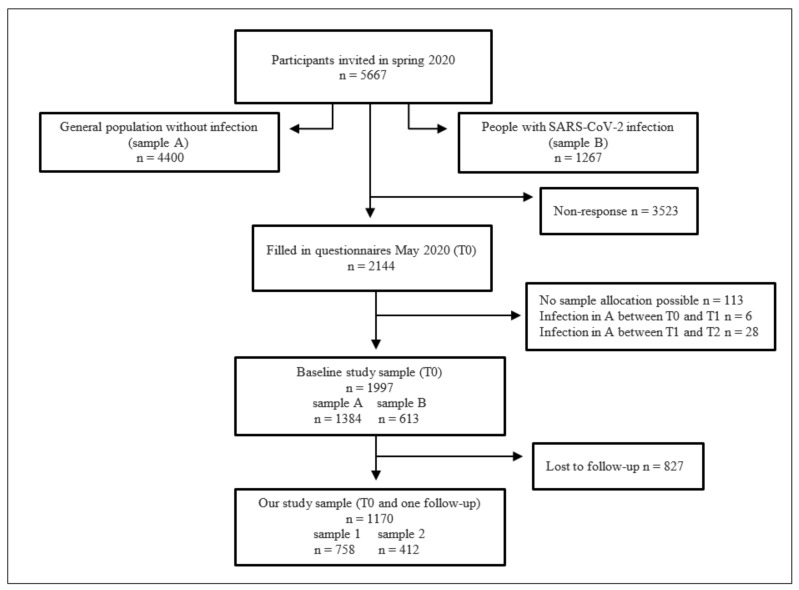
Flowchart of baseline sample recruitment and response and the final sample for our analysis.

Participants in both samples could choose to fill out the questionnaire either online or using a paper version. Survey assessments took place in May 2020 (T0), November 2020 (T1) and July 2021 (T2). T0 was the time towards the end of the first pandemic wave and first lockdown in Germany, T1 dated the beginning of the second lockdown and T2 was a few months after the end of the second lockdown in Germany. Figure 1 shows the originally recruited study participants as well as the dropouts based on the different measurement time points. Subjects who self-reported infection with SARS-CoV-2 between T0 and T1 or T1 and T2 were excluded.

### 2.3. Measures

A short version of the Patient Health Questionnaire, the PHQ-4 [21], was used to measure self-reported depression and anxiety symptoms on a scale ranging from 0 (not at all) to 3 (nearly every day) (PHQ-4 at baseline, McDonald’s omega, ω = 0.84). Here, two items measure the symptoms of anxiety (GAD-2), and two items measure depressive symptoms (PHQ-2). The mean of all four items at the respective time of measurement was used for all analyses. The sum score of PHQ-4 > 6 indicates clinically relevant symptoms; this corresponds to a mean value of >1.5.

Perceived stress was measured with the Perceived Stress Questionnaire (PSQ) with 20 items [22,23] on a scale ranging from 1 (nearly never) to 4 (most of the times). One sum score was calculated summarizing all 20 items (PSQ at baseline, McDonald’s omega, ω = 0.93), resulting in the range of 20 to 80. Moreover, four sum scores were calculated for the subcategories *worry*, *tension*, *demands and joy*.

## 3. Results

The sample for our analyses includes only those participants that reported values in the baseline measurement (May 2020) and at least in one of the two follow-up measurements (November 2020 and July 2021). This leads to a final sample consisting of *N* = 1170 participants at baseline (T0), from which *N* = 412 reported a SARS-CoV-2 infection before baseline and *N* = 758 participants had no known SARS-CoV-2 infection.

### 3.1. Sample Characteristics at Baseline

Table 1 shows demographic characteristics, means and standard deviations for the final sample without (sample 1) and with a SARS-CoV-2 infection (sample 2). In the sample of people with SARS-CoV-2 infection, *N* = 511 (83.4%) were treated at home, *N* = 79 (12.9%) in hospital and *N* = 29 (4.7%) needed ICU admission. In both groups, the most common chronic conditions were cardiovascular disease (16.8%), metabolic disease (11.9%) and musculoskeletal disease (11.7%). In total, 81 participants (4.1%) reported a history of mental illness. There were no differences between both groups. 

### 3.2. Prediction of Depressive and Anxiety Symptoms over Time (Model 1)

First, we predict the *PHQ-4* using *time*, *sample*, *education* and *pre-existing disease*. The model’s total explanatory power is substantial (conditional R^2^ = 0.72), and the part related to the fixed effects alone (marginal R^2^) is 0.02. 

The effect of time is statistically significant and positive (*p* < 0.001; Std. beta = 0.07), and the effect of time^2^ is statistically significant and negative (*p* = 0.010; Std. beta = −0.03). These effects of time indicate that depressive and anxiety symptoms increased from May 2020 to November 2020 and seemed to stabilize until July 2021 (displayed in Figure 1). 

**Figure 2:**
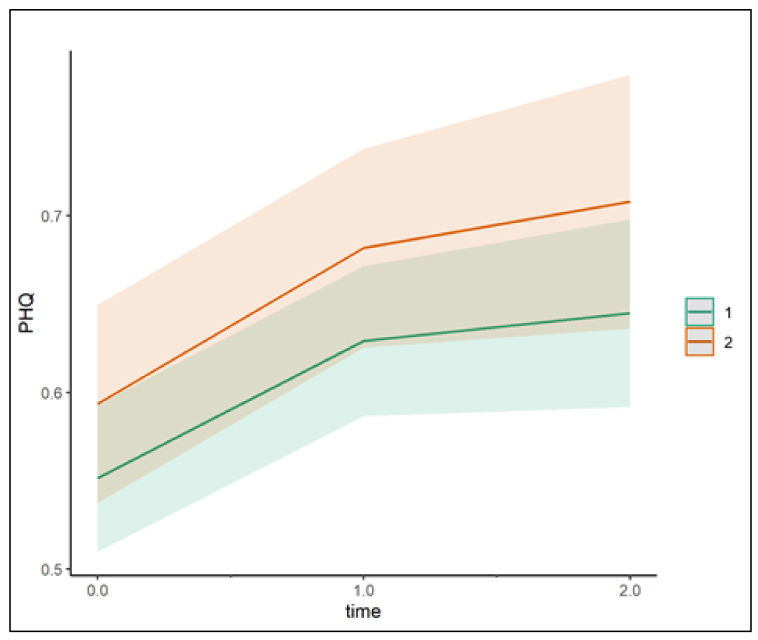
Changes in depressive and anxiety symptoms over time (model 1). Note: PHQ: mean values of PHQ-4; sample 1 = individuals without SARS-CoV-2 infection; sample 2 = individuals after a SARS-CoV-2 infection.

Depressive and anxiety symptoms seem, on average, to be higher for people who had a SARS-CoV-2 infection compared with those without (see Table 1 and Figure 2). However, this difference between both samples is not statistically significant in model 1. 

The effect of education on depressive and anxiety symptoms is statistically significant and negative (*p* < 0.001; Std. beta = −0.09), indicating that people with higher education reported lower depressive and anxiety symptoms. Moreover, the effect of pre-existing chronic disease is statistically significant and positive (*p* = 0.033; Std. beta = 0.12), indicating that people with pre-existing disease reported higher depressive and anxiety symptoms.

### 3.3. Prediction of Depressive and Anxiety Symptoms over Time When Integrating General Stress as a Predictor (Model 2)

In model 2, the model’s total explanatory power is substantial (conditional R^2^ = 0.77), and the part related to the fixed effects alone (marginal R^2^) is 0.55. 

Other than in model 1, after integrating stress in the form of the PSQ as a predictor of depressive and anxiety symptoms in model 2, the depressive and anxiety symptoms did not display time dependency. The same as in model 1, the effect of the sample on depressive and anxiety symptoms is statistically non-significant in model 2. The effect of stress in the form of the PSQ is statistically significant and positive (*p* < 0.001; Std. beta = 0.73). These results suggest that increases in depressive and anxiety symptoms might be mediated via perceived stress. In other words, an increase in perceived stress might explain the increase in depressive and anxiety symptoms.

Like in model 1, the effect of education is statistically significant and negative (*p* < 0.001; Std. beta = −0.10), and the effect of disease is statistically significant and positive (*p* = 0.011; Std. beta = 0.08). 

### 3.4. Prediction of Depressive and Anxiety Symptoms over Time, with Detailed Analysis of the Four Subcategories of Stress (Model 3)

When adding subcategories of stress, model 3, the model’s total explanatory power is substantial (conditional R^2^ = 0.76), and the part related to the fixed effects alone (marginal R^2^) is 0.64. 

Again, the effect of time, time^2^ and the sample is statistically non-significant. The subcategories of stress, *worry* (*p* < 0.001; Std. beta = 0.46) and *tension* (*p* < 0.001; Std. beta = 0.29) emerged as statistically significant and positive predictors of depressive and anxiety symptoms. Higher worries, in particular, strongly predict higher depressive and anxiety symptoms in people in both samples. *Joy* emerged as a statistically significant and negative predictor (*p* < 0.001; Std. beta = −0.15), indicating that people experiencing higher joy experienced lower depressive and anxiety symptoms. Contrary to what might be assumed, the effect of *demands* emerged as statistically significant and negative (*p* < 0.001; Std. beta = −0.09), which would indicate perceiving higher demands is associated with lower depressive and anxiety symptoms. However, compared to the other predictors, this effect of demands on depressive and anxiety symptoms was relatively small and should be interpreted cautiously. 

Similar to model 1 and model 2, the effect of education is statistically significant and negative (*p* < 0.001; Std. beta = −0.05). The effect of disease is statistically non-significant. However, the interaction effect of the subcategory *worry* and previous disease is statistically significant and positive (*p* = 0.030; Std. beta = 0.05). This indicates that participants with a pre-existing disease who had higher ratings in *worry* also reported higher depressive and anxiety symptoms, compared to participants without pre-existing diseases.

All the results of models 1 to 3 are summarized in Table 2.

## 4. Discussion

The results show that symptoms of depression and anxiety (measured with PHQ-4) increased during the first year of the pandemic in the general population and in individuals who were infected with SARS-CoV-2 early in the pandemic. However, this increase remained relatively small, compared with PHQ-4 norm data [7]. Although depression and anxiety seemed on average a little higher in people after SARS-CoV-2 infection compared to persons who did not report an infection, these differences were not significant. 

Past studies in several countries have already shown that stress increased during the pandemic [13,15]. In addition, our results indicate that stress (measured with PSQ) emerged as a strong predictor of the trajectories of depressive and anxiety symptoms during the first waves of the COVID-19 pandemic. Our results suggest that stress is a mediator of depressive and anxiety symptoms during the pandemic because, when including measures of stress in our model, the increases in depressive and anxiety symptoms from May 2020 to November 2020 and July 2021 were not significant. In detail, more stress in the form of worry, in particular, seemed to be associated with higher depression and anxiety symptoms, whereas joy seemed to buffer those mental health symptoms. 

Moreover, those with a pre-existing disease regarding mental and/or physical health and lower education reported higher levels of depression and anxiety. In detail, the results indicated that particularly those with a pre-existing disease who experienced more stress in the form of worry also reported higher levels of depressive and anxiety symptoms. This is in line with the indications of other studies [6,9] that vulnerable groups in times of COVID-19 are those with lower socioeconomic status and poorer health symptoms.

Stress is one of the leading causes of mental health problems and disorders [24,25]. Studies suggest that in times of COVID-19 it might be an important mediator between risk perception and symptoms of depression and anxiety [26]. Thus, if stress can be effectively reduced, for example, by sufficient coping, depressive and anxiety symptoms could also be alleviated. There are several ways to prevent or reduce stress, including exercise, relaxation techniques, healthy lifestyle and social support. Due to contact restrictions, social support was limited during the pandemic. In favor of stress prevention, ways should be found to enable social contacts as well as make them possible even during a crisis situation.

Our finding that people with pre-existing disease are at increased risk of mental health problems is consistent with other studies [27,28]. Therefore, in times of pandemic, a special focus of prevention should be on this vulnerable group. This is particularly important since pronounced mental symptoms such as avoidance behavior in the context of an anxiety disorder as well as depressive and anxiety symptoms like lack of energy or tiredness can lead to a worsening of the underlying disease due to non-observance of medical contacts or neglectfulness of medical treatment.

### Limitations

This study used only a short measure for depressive and anxiety symptoms with the four items of PHQ-4. However, past studies indicate high correlations between the longer versions of the PHQ and the PHQ-4 [21]. Also, the concepts of depression and anxiety partly overlap in the used instrument; still, we did not attempt to separate the components. The self-reporting nature of our measures regarding stress and depressive and anxiety symptoms could have been sensitive for the under- or over-reporting of symptoms. Self-reporting instruments rely on individuals’ subjective perceptions and experiences, introducing the potential for bias in reporting. Factors such as social desirability and varying levels of introspection among respondents can influence the accuracy of self-reported data. Additionally, individuals may under-report or over-report their symptoms due to stigma, fear of judgment or the desire to conform to societal norms. Therefore, future studies should also use the ratings of health professionals to corroborate findings and enhance the validity and reliability of research outcomes in the realm of stress and mental health assessment. It is possible that during the course of this study further SARS-CoV-2 infections occurred in either sample, but participants were not aware of them or did not report them. Furthermore, our study is not free from potential selection bias. Among those initially invited, less than 50% participated; in addition, there was some dropout in both groups. Fortunately, our analysis indicated that loss of follow-up was non-differential and therefore did not lead to selection bias. Apparently, the participants of this study had a high educational status; however, we could not quantify the extent of the resulting bias. 

Another limitation of this study pertains to our inability to incorporate symptom severity of acute infection as a predictor for participants who reported SARS-CoV-2 infection. While we acknowledge the potential influence of symptom severity on mental health outcomes, this variable could not be included due to the absence of individual symptom-level data. Also, the duration between the infection and the baseline questioning could influence the results. Unfortunately, we cannot specify the infection dates from the data, but the baseline measurement took place shortly after the first registered SARS-CoV-2 infection in Germany. Furthermore, some of those infected could have developed long COVID symptoms, i.e., their worse trajectory was not linked to the initial experience of infection but rather to continuing symptoms. Given the fact that the risk of post-COVID-19 conditions was rather high for the wild variant addressed in this study [29], a non-negligible group could have been affected. 

## 5. Conclusions

Our results show no differences in depressive and anxiety symptoms between individuals who experienced SARS-CoV-2 infection early in the pandemic and those not infected during the course of the pandemic. Overall, depressive and anxiety symptoms increased during the first months of the pandemic in 2020 and seemed to stabilize from Dec 2020 to July 2021. In particular, stress in the form of worry emerged as a strong predictor of depressive and anxiety symptoms. Mental health interventions that reduce stress, and especially worry, could promote resilience in future crises. Future studies should target those interventions at vulnerable groups that suffer more from their worries, such as those with lower socioeconomic status and poorer health.

## Figures and Tables

**Table 1:** Sociodemographic characteristics at baseline of the two samples: those not reporting a SARS-CoV-2 infection during the study (sample 1) and those who experienced SARS-CoV-2 infection early in the pandemic (and before the start of the study (sample 2)).

	Sample 1 *M* (*SD*)	*N*	Sample 2 *M* (*SD*)	*N*
Age (T0)	45.69 (15.97)	756	45.80 (15.66)	400
Gender (female; T0)	54.9%	758	51%	412
Education (T0)				
*No professional qualification*	37 (5.1%)	758	17(4.1%)	412
*Professional qualification*	236 (31.1%)	758	118 (28.6%)	412
*University degree*, *Bachelor*	91 (12%)	758	44 (10.7%)	412
*University degree*, *Master/Diploma*	364 (48.5%)	758	217 (54.7%)	412
Pre-existing disease % (T0)	38.7%	293	39.1%	161
PHQ-4 baseline (T0)	0.56 (0.54)	758	0.59 (0.67)	411
PHQ-4 follow-up (T1)	0.62 (0.59)	758	0.69 (0.67)	412
PHQ-4 follow-up (T2)	0.63 (0.63)	465	0.65 (0.69)	233
PSQ baseline (T0)	32.3 (19.12)	737	32.47 (18.83)	393
PSQ follow-up (T1)	36.20 (19.45)	717	38.43 (20.29)	398
PSQ follow-up (T2)	36.20 (20.81)	465	37.26 (20.56)	231

*M*—mean; *SD*—standard deviation; PHQ-4—Patient Health Questionnaire-4 PSQ—Perceived Stress Questionnaire; T0 baseline May 2020; T1 November 2020; T2 July 2021. Note: sample includes only those that participated at least in baseline (T0) measurement and one follow-up measurement.

**Table 2:** Variables associated with depressive and anxiety symptoms (PHQ-4) over the course of the study.

				Confidence Intervals Std. Beta		
**Model 1**						
	beta (B)	Std. beta (β)	Std. error	CI lower	CI upper	*p*
Time	0.11	0.07	0.02	0.04	0.10	<0.001
Time^2^	−0.03	−0.03	0.01	−0.06	−0.01	0.010
Sample	0.05	0.09	0.03	−0.02	0.20	0.170
Education	−0.06	−0.09	0.01	−0.14	−0.04	0.001
Disease	0.07	0.12	0.03	0.02	0.23	0.033
Sample × Time	0.01	0.01	0.02	−0.04	0.06	0.728
**Model 2**						
	beta (B)	Std. beta (β)	Std. error	CI lower	CI upper	*p*
Time	−0.04	−0.02	0.02	−0.04	0.01	0.117
Time^2^	0.01	0.01	0.01	−0.01	0.04	0.207
Sample	0.02	0.03	0.02	−0.03	0.10	0.319
Education	−0.06	−0.10	0.07	−0.13	−0.07	<0.001
Disease	0.05	0.08	0.02	0.02	0.15	0.011
General stress (PSQ)	0.02	0.73	0.04	0.70	0.76	<0.001
Sample × Time	0.02	0.01	0.02	−0.05	0.03	0.727
**Model 3**						
	beta (B)	Std. beta (β)	Std. error	CI lower	CI upper	*p*
Time	−0.03	−0.00	0.02	−0.03	0.02	0.239
Time^2^	0.02	0.01	0.01	−0.01	0.04	0.202
Sample	0.01	0.03	0.02	−0.03	0.08	0.559
Education	−0.03	−0.05	0.06	−0.08	−0.02	<0.001
Disease	−0.02	0.04	0.01	−0.02	0.09	0.508
Worry	0.01	0.46	0.05	0.44	0.50	<0.001
Tension	0.01	0.29	0.06	0.24	0.33	<0.001
Joy	−0.00	−0.15	0.05	−0.19	−0.12	<0.001
Demands	−0.00	−0.09	0.04	−0.12	−0.06	<0.001
Sample × Time	0.00	0.00	0.04	−0.04	0.04	0.815
Disease × Worry	0.01	0.05	0.02	0.00	0.10	0.030

PSQ—Perceived Stress Questionnaire. Note: *N* = 758 in the general population and *N* = 412 in SARS-CoV-2-infected patients.

## Data Availability

The data presented in this study are available upon request from the corresponding author. The data are not publicly available for reasons of data privacy.
